# Evaluation of different inflammatory markers during the infection of domestic cats (*Felis catus*) by *Cystoisospora felis* (Coccidia: Apicomplexa)

**DOI:** 10.1186/s12917-024-04295-2

**Published:** 2024-11-16

**Authors:** Marwa M. Attia, Sara S. Barsoum, Hanadi B. A. Baghdadi, Olfat A. Mahdy, Sohila M. EL Gameel

**Affiliations:** 1 Department of Parasitology, Faculty of Veterinary Medicine, Cairo University, Giza, 12211 Egypt; 2 Zoology Department, Faculty of Science, Fayoum University, Fayoum, 63514 Egypt; 3 Biology Department, College of Science, Imam Abdulrahman Bin Faisal University, Dammam, 31441 Saudi Arabia

**Keywords:** Intestinal parasites, *Coccidia* of cats, Stress markers, *Cystoisospora felis*

## Abstract

**Background:**

*Cystoisospora felis* or *Isospora felis* is a ubiquitous apicomplexan protozoon parasite infecting domestic cats worldwide.

**Objectives of the study:**

this study aims to identify the causative agent of diarrhea in cats by determining several elevating stressors caused by these coccidian protozoans with molecular characterization. So, from January 2023 to April 2023, a total of 370 domestic cats were hospitalized at various clinics in the Cairo and Giza Governorates. Fecal samples were taken from these animals and examined by concentration floatation techniques using a saturated salt solution. The positive samples were sporulated to identify the collected oocyst. Venous blood was taken from the infected cats to evaluate the associated oxidative stress marker (lipid peroxidation products (MDA).

**Results:**

Out of 370 examined domestic cats, 27(7.29%) were positive for *C. felis.* The MDA levels increased with age, and females were higher than males. DNA was extracted from fecal samples for amplification of the ITS1 gene, followed by sequencing. The ITS1 gene was amplified and showed bands at 224 bp. The partial nucleotide sequence of the ITS1 gene was aligned with the reference sequences.

**In a conclusion:**

*C. felis* increases the free radicals, which in turn means the animals have stress and need a schedule to treat these animals with new, safe protocol drugs that give no resistance and are highly efficient.

## Introduction

*Cystoisospora felis* or *Isospora felis* is a ubiquitous apicomplexan protozoon parasite infecting domestic cats worldwide [1, 2] *Isospora felis* was the name given by [3] **Wenyon**, **1923** to the coccidia with enormous oocysts, and he also characterized several of its endogenous phases in the cat’s gut. Based on the size of the oocysts [3, 4], **Wenyon (1923**, **1926)** was the first to recognize that there were at least three different types of coccidia in cats: (a) large oocyst size measuring about 40 μm; (b) medium oocyst size measuring around 25 μm; and (c) a tiny– sized oocyst with about 10–12 μm long. In 1970–1980, it was discovered that the species of the coccidia had medium and big-size oocysts in cats and dogs that showed high specificity [5, 6]. The enormous ovoid oocysts of *C. felis*, which measure 32–53 × 26–43 μm in fecal specimens, simplify the species’ identification. Sporulation occurs a few hours after the excretion of oocysts in the environment after non-sporulated oocysts have been shed [5] .

The feline coccidian is not contagious; before the discovery of these parasites’ life cycles, these parasites were known by different names, including *Isospora cati* [6], *Isospora rivolta*, and *Isospora gondii* [7]. For parasites that are similar to *Isospora rivolta* that infect cats, coccidia had a direct fecal-oral cycle. In cats and mice, *C. felis* and *C. rivolta* were discovered in extra-intestinal stages by **Frenkel and Dubey** [8]. Rodents were reported to harbor an encysted stage (tissue cyst) of *C. felis* and *I. rivolta* [8, 9].

Other hosts, such as mice, can serve as paratenic or transport hosts since the parasite persists but does not reproduce. Histological and bioassay tests revealed that *C. felis* can survive in mouse tissues for up to two years. A substantial cyst wall encloses a solitary parasite [2, 8, 10]. For cats, even being in the most sanitary conditions can contract *C*. *felis*. Thus, how it spreads is unknown [11]. Adults probably feline produce just a few oocysts, which can infect young kittens. For *C. felis*, transmission from persistently infected individuals has been proposed through the placenta or milk secretion [11]. The only known method of transmission is post-natal parasite ingestion from the environment (by eating oocysts or contaminated tissues). Newborn cats were susceptible to *C. rivolta*, while weaned cats showed some resistant [2].

After feeding weaned cats sporulated oocysts [12], **Shah (1971)** described in detail the asexual and sexual cycle of *C. felis*. Schizonts were described with three generations that were morphologically unique in merozoites.

According to [12] **Shah (1971)** the parasite was found in the distal part of the intestinal villus in cats that had shed *C. felis* oocysts. *C. felis* proliferated in enterocytes throughout the villus, and meronts were discovered near the base of the villus, even in light infections during the cycle’s early stages; [13].

In cats, *C. felis* pathogenicity is the subject of conflicting reports. Severe diarrhea has been noted in *C. felis-*infected cats in some accounts, particularly before the availability of enteric virus prophylaxis [14]. Because *C. felis* divided on the surface of epithelial enterocytes (which have a rapid turnover rate) as opposed to *C. rivolta*, which can divide in glands of Lieberkühn (germinal cells that affect enterocyte replication), *C. rivolta* was more pathogenic than *C. felis* [2].

Coccidiosis in young kittens is rare. Young animals in settings with a large population and possible contamination are typically affected. Illness may become an issue in catteries and kennels used for breeding or boarding. Nearly all cats contract *C. felis* at some point. The majority of cats contract the infection when they are young, and when clinical symptoms do appear, they usually do so in kittens and pups (4–12 weeks) and very rarely in adult canines who are immunocompromised or crippled. Illness in kittens usually arises during the stress of weaning. Clinical symptoms appear in puppies approximately two weeks following infection. Immunity is good following oocyst exposure, particularly in cats [15].

Infections in cats typically show no symptoms in most cases. For several animals, diarrhea is the most noticeable clinical symptom. There is occasional diarrhea linked to acute instances. Severe *C. felis* infections in kittens can cause mucoid or watery diarrhea; later on, bloody diarrhea is rare; further symptoms may include weight loss, anorexia, dehydration, and abdominal pain. Anemia, respiratory, and neurological symptoms are rare [9, 16].

In case of parasitic infestations, the body’s immune system produces higher levels of reactive oxygen species (ROS) as nitric oxide, superoxide radical (O2.−), hydrogen peroxide (H2O2), and hydroxyl radical (^•^OH) than usual [17]. Because it is used as a fundamental defense mechanism in the host’s resistance to infection with parasites, as in the case of sheep infections with *D. dendriticum* [18] and liver fluke [19]. Antioxidants are produced by host cells to neutralize ROS’s harmful effects. As Superoxide dismutase (SOD) converts the superoxide anion (O2) into hydrogen peroxide [20], which is subsequently detoxified by catalase (CAT) and glutathione peroxidase (GPx), with the final product of the process being the water molecule. However, the excessive generation of (ROS) than antioxidants causes a case of oxidative stress [21]. Unchecked ROS overproduction causes protein and lipid peroxidation and DNA strand damage, which harms cells and causes them to die. One well-known indicator of serum lipid peroxidation is malondialdehyde (MDA) [22].

So, this study aimed to identify the causative agent of diarrhea in cats, determining several elevating stressors caused by these coccidian protozoan parasites in domestic cats and their effect on MDA level as one of the oxidative stress markers.

## Materials and methods

### Collection of samples

From January 2023 to April 2023, 370 domestic cats (175 males and 195 females) with severe diarrhea were examined at various Cairo and Giza Governorates clinics. The cats were between 6 months and 2.5 years old. Fecal samples and EDTA whole blood samples were collected and sent to the Parasitology Department, Faculty of Veterinary Medicine, Cairo University, for further analysis [23, 24]. The Institutional Animal Care and Use Committee Vet. *CU. IACUC*,* with the number Vet Cu 03162023728*,* approved this study.* All data for animals were recorded for age and sex.

### Fecal analysis

Fecal samples were taken from these animals and examined by concentration floatation techniques using a saturated salt solution [25]. Fecal samples were analyzed to detect any parasitic other than *C. felis* using concentration floatation techniques as described by [15, 26, 27]. For oocyst sporulation, the positive samples for oocyst were put in separate vials with a solution of potassium dichromate (K2Cr2O7) at a concentration of 2.0% (w/v) then incubated at room temperature for ten days and at four ^°^C for extended storage [28].

### Collection of blood

The anti-coagulated blood was used to prepare a thin blood film and stained with Giemsa stain to be examined for the presence of any blood parasites.

### Measurement of oxidative stress markers (MDA)

Five milliliters of venous blood were drawn from infected and non-infected cats in tubes devoid of EDTA. The blood was centrifuged at 3500 rpm for 15 min. The serum was extracted from the clotted blood and stored at -20 °C until it was examined [29–31]. According to [32] **Placer et al. 1966**, the amount of malondialdehyde (MDA) in sera (both the positive and negative sera) was measured [17, 33].

### DNA extraction

DNA was isolated from fecal samples collected from five cats found to be infected with *C. felis* using a manual commercial kit (Thermo Scientific Gene JET Genomic DNA Purification Kit; USA), according to the manufacturer’s instructions. DNA was stored at − 20 °C until used in DNA amplification.

### Polymerase chain reaction amplification

Polymerase chain reaction (PCR) analysis was performed using the forward primer 5`- CTACTGAATCCCATAATCAGGAC-3` and reverse primer 5`- CCAAAATCTCAAGGAGAGAGGAG-3`. Primers with standard PCR procedures for amplifying the *C. felis* ITS1 gene as [34] **Scorza et al.**,** 2019** described. Amplification was performed in a programmable thermal cycler (Nexus Gradient; Eppendorf, Germany). The following PCR protocol was used: 95 °C for 10 min followed by a set of 50 cycles, 95 °C for 20 s, 55 °C for 20 s, and 72 °C for 20 s with melt curve analysis to verify the specificity and identity of the PCR products according to. The corresponding amplicons were checked on a 1.5% agarose gel. After gel staining with ethidium bromide, the DNA bands were visualized using ultraviolet transillumination (0.5 mg/ml).

### Sequencing of PCR product

PCR products were purified using the Big Dye X Terminator Purification Kit (Thermo Fisher Scientific, Waltham, MA) according to the manufacturer’s instructions. Cycle sequencing was performed using the Big Dye Terminator version 3.1 Cycle Sequencing Kit (Thermo Fisher Scientific). The samples were injected into a 3500 Genetic Analyzer (Thermo Fisher Scientific). DNA sequencing was performed in both directions using the forward and reverse primers for the ITS1 nuclear gene. Nucleotide sequences were first analyzed and edited to check electropherogram quality using Finch TV software version 1.4.0 (Geospira Inc., Seattle, WA). Nucleotide sequence analysis was performed using the Basic Local Alignment Search Tool (BLAST; blast.ncbi.nlm.nih.gov). The obtained DNA sequences were subsequently aligned and compared with verified sequences of *C. felis* strains available in GenBank using Clustal W and Bioedit software. *T. gondii* and *Neospora caninum* were used as outgroup species. The phylogenetic tree was constructed using the software program MEGA 7. The sequences analyzed in this study were finally deposited in the GenBank.

## Results

**Fig. 1:**
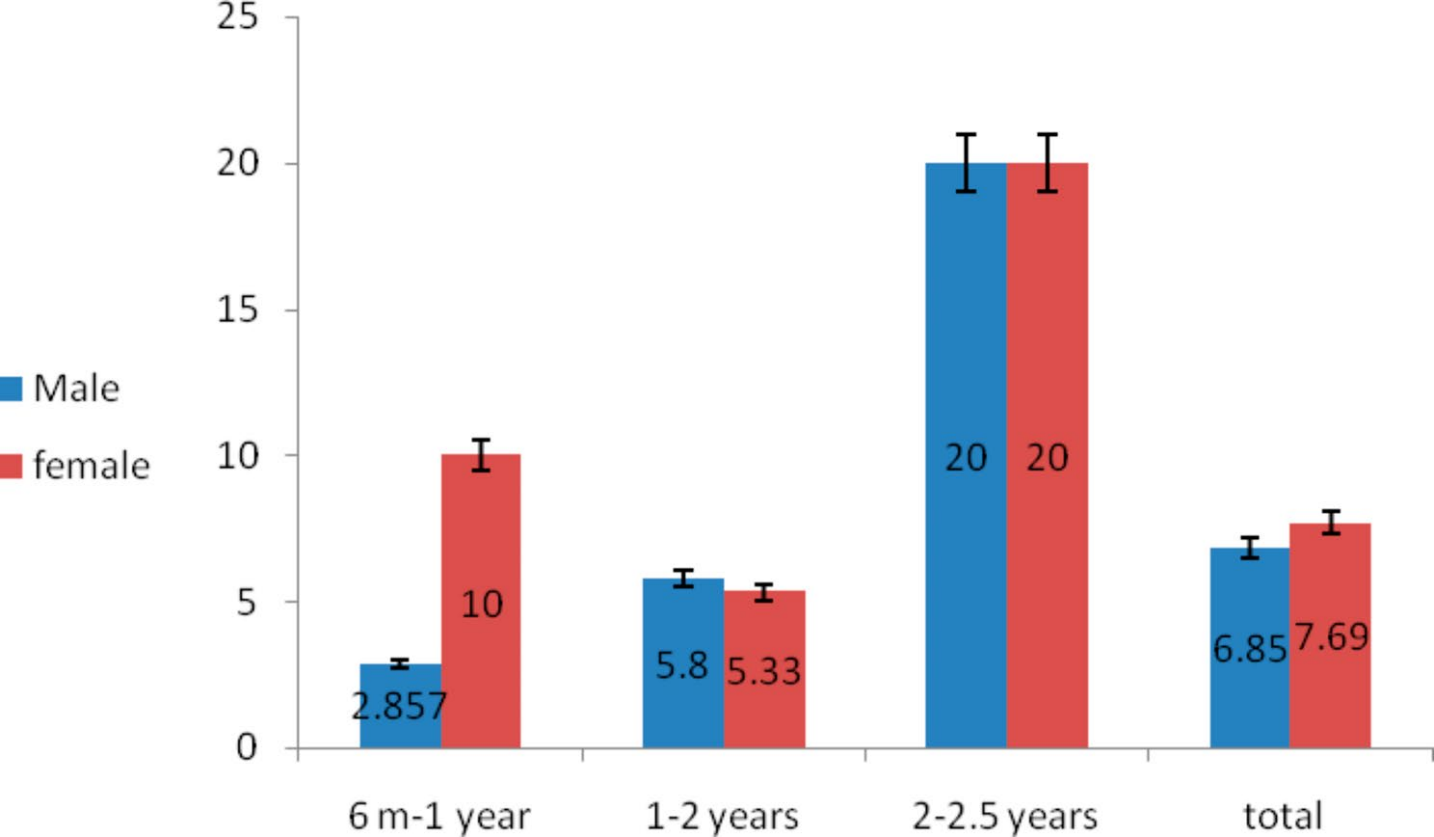
Prevalence study of *C. felis* in relation to sex and age in domestic cats; error bars refers to standard errors

Of 370 examined domestic cats, 27 (7.29%) were positive for *C. felis.* According to sex 12/175 males (6.86%) and 15/195 females (7.69%), their fecal samples showed *C. felis* oocysts. The age groups were classified into three groups: from 6 months to 1 year, 1/35 male (2.85%) and 2/20 female (10%) were infected. The age group (> one year to 2 years) was 7/120 males (5.8%), and 8/150 females (5.33%) were infected. While in groups > 2 years − 2.5 years, 4/20 males (20%) and 5/25 females (20%) were infected. (Table 1; Fig. 1).

**Table 1:** Prevalence study of *C. felis* in relation to sex and age in domestic cats

Sex Age	Male cats		Female cats	
	Examined	Positive	Examined	Positive
6 Months − 1 year	35	1(2.857%)	20	2(10%)
> 1 year to 2 years	120	7(5.8%)	150	8 (5.33%)
Over 2 years − 2.5 years	20	4(20%)	25	5(20%)
Total	175	12(6.86%)	195	15(7.69%)

**Fig. 2:**
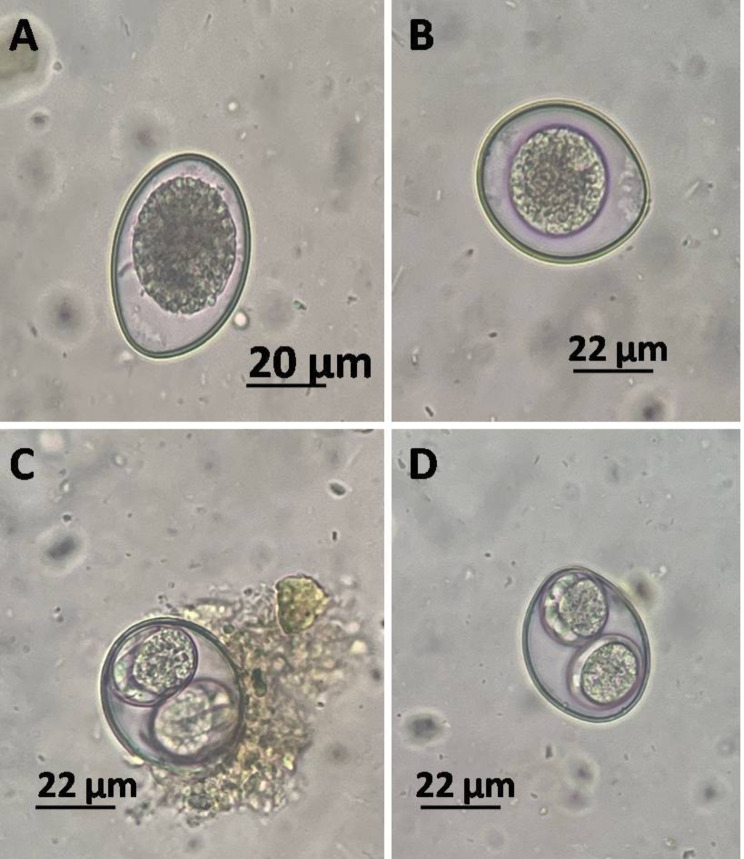
*C. felis* collected from domestic cats feces. **A; B**: unsporulated oocyst with smooth wall and elongate-ovoid shape, with 45–59 μm X 29–45 μm. **C; D**: sporulated oocyst with two sporocysts, each with four sporozoites, and sporocyst residuum were compact masses

The oocysts are elongate-ovoid in shape, measuring 45–59 μm X 29–45 μm, and have smooth walls. Two colorless sporocysts were included in the sporulated oocyst, and the four sporozoites in the sporocyst residuum were compact masses of small granules. There was no oocyst residue, polar granule, or micropyle. The bodies at the site and subsite were absent. (Fig. 2**).**

The oxidation of lipids (MDA) increases statistically with age and sex; the female is statistically higher than the male (Table 2). The age groups were classified into three groups: 6 months-1 year. All the values were statistically higher than those in control healthy domestic cats of the same age and sex.

**Table 2:** Oxidative stress marker (Malondialdehyde) in different age and sex in examined domestic cats infected with *C. felis.* Measurement unit/ µmol/l; data expressed as minimum- maximum (mean ± standard errors)

Sex Age	Male cats	Female cats	Normal control negative cats	
			Male	Female
*6 Months − 1 year*	2.98–3.35 (3.165 ± 1.5*)	2.58–3.850 (3.215 ±1.3**)	1.450–3.200	1.440–1.559
*> 1 year to 2 years*	3.48–5.35 (4.415 ± 0.65**)	3.689–5.889 (4.789 ± 0.55***)	1.460–3.250	1.580–1690
*> 2 years − 2.5 years*	3.58–5.65 (4.615 ± 0.55***)	3.990–5.999 (4.994 ± 1.55***)	1.687–3.249	1.780–1.980

* *P* < 0.05; ** *P* < 0.01; *** *P* < 0.001

The ITS1 gene was sequenced to identify the *C. felis* genotypes. The partial nucleotide sequences of the ITS1 gene of the five isolates were aligned with the reference sequences using BLAST. All sequences were compared with the identified sequences of *C. felis* species using the MEGA7 program for designing the phylogenetic trees, which confirmed the preliminary results obtained by the BLAST search tool.

**Fig. 3:**
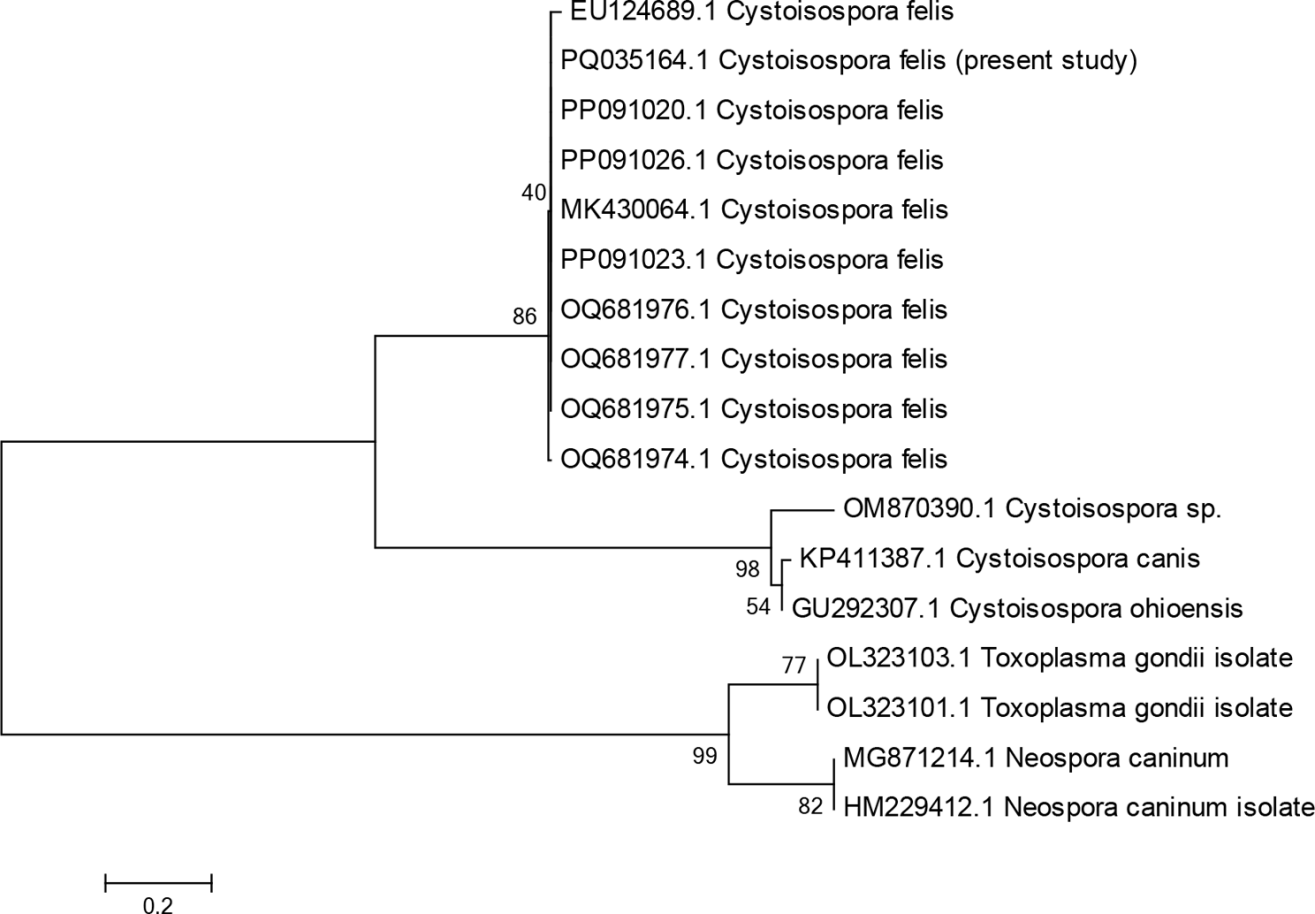
Genetic relationship between *C. felis* isolate (accession no.: PQ035164.1) and the reference sequences based on phylogenetic analysis of ITS1 nucleotide sequences on GenBank

The partial nucleotide sequences of the ITS1 gene of all *C. felis* (GenBank sequence accession number: PQ035164.1 shared 100% homology with the reference partial nucleotide sequence of *C. felis* on GenBank (PP091026.1, PP091020.1, and MK430064.1); Fig. 3.

## Discussion

Three sporulated oocysts were morphologically observed to be infected domestic cats, as recorded in [2]. The sporulated oocysts held two sporocysts lacking a Stieda body, each containing four sporozoites and exhibiting characteristics of the *Cystoisospora* genus; [35].

The morphology of coccidian species should be thoroughly compared to other species within the host family that share similar characteristics. Tenter et al. [36] was reviewed host-related criteria morphological and molecular characteristics to be considered when classifying coccidia. To compare the physical traits of otter oocysts with those of other *Cystoisospora* spp. It was previously reported in carnivores of the family Mustelidae.

In addition to the feline definitive host, both feline *Isospora* species exhibit extraintestinal stages in a range of paratenic hosts. Oocysts are excreted by 27(7.29%) of the cats studied. Stray cats and domestic cats more frequently excrete oocysts. It is not believed that cat coccidiosis is a widespread issue and is typically only observed in naturally infected kittens, where additional disease-causing agents may be present.

In the small intestine and sporadically in the cecum, *C. felis* grows in enterocytes. There are three distinct structural forms of asexual phases. Endodyogeny is the initial asexual division. Oocysts are secreted for around 11 days during the prepatent phase, which is between 7 and 11 days. There may be slight microscopic alterations like congestion, degradation of the surface enterocytes, and neutrophil infiltration. The most vulnerable kittens are those that are four weeks old, and 10^5^ oocysts can cause enteritis, emaciation, and death [9, 37].

Cats become immune to *C. felis* because, following infection, they produce fewer or no oocysts when exposed to *C. felis* oocysts. According to studies, cats naturally infected with *C. felis* have lower antibody titers than cats that are experimentally inoculated with the parasite [37].

Cell structure and function become disorganized when the stress signals caused by polyunsaturated fatty acids in membrane phospholipids—the primary target substrates for free oxygen radical activity—are altered [38, 39].

The deactivation and removal of ROS need the action of antioxidant defense systems, which comprise the enzymes glutathione peroxidase (GPX), catalase (CAT), and superoxide dismutase (SOD). Reactive oxygen species (ROS) can be neutralized by vitamins A, C, and E, examples of non-enzyme antioxidant defense [31].

Free radical production is a complication of numerous diseases affecting various organ and tissue systems; [40, 41].

Antioxidant defense mechanisms are activated to modify and combat the production of free radicals and neutralize their adverse effects. Oxidative stress happens when the equilibrium between ROS formation and antioxidant activity is upset, aggravating the underlying illness.

The results of this study showed that MDA increases with age and is higher in females than in males for a variety of reasons, including the fact that SOD, the body’s first line of defense against ROS and the catalases, was the detoxification of superoxide radicals (O2); [42, 43]. When polyunsaturated fatty acids are broken down by ROS, a process known as lipid peroxidation occurs, creating damaging compounds like malondialdehyde (MDA).MDA is a highly reactive aldehyde that harms tissues by oxidation. MDA is a significant thiobarbituric acid reactive compound (TBARS) in the organism and is a member of the TBARS class. As a result, the TBARS measurement is frequently employed to determine the MDA concentration for the benefit of technique simplicity. The two most significant indicators of oxidative stress and lipid peroxidation in biological samples are TBARS and MDA, respectively (Aguilar et al., 2020). In conclusion, *C. felis* increases the free radicals, which in turn means the animals have stress and need a schedule to treat these animals with new, safe protocol drugs that give no resistance and are highly efficient.

## Data Availability

All the authors declare that all the data supporting the results reported in our article which were found included in this article only.
